# Efficacy of Tyrosine Kinase Inhibitors in ALK and EGFR-Mutated Non-Small Cell Lung Cancer with Brain Metastases

**DOI:** 10.3390/medsci13030200

**Published:** 2025-09-18

**Authors:** Walid Shalata, Rashad Naamneh, Wenad Najjar, Mahmoud Abu Amna, Mohnnad Asla, Abed Agbarya, Ronen Brenner, Ashraf Abu Jama, Nashat Abu Yasin, Mhammad Abu Juda, Ez El Din Abu Zeid, Keren Rouvinov, Alexander Yakobson

**Affiliations:** 1 The Legacy Heritage Cancer Center, Dr. Larry Norton Institute, Soroka Medical Center, Beer-Sheva 8410501, Israel; 2 Goldman Medical School, Faculty of Health Sciences, Ben-Gurion University of the Negev, Beer-Sheva 8410501, Israel; 3 Neurosurgery Department, Soroka Medical Center, Beer-Sheva 8410501, Israel; 4 Neurosurgery Department, Hadassah Hebrew University Medical Center, Jerusalem 91200, Israel; 5 The Ruth and Bruce Rappaport Faculty of Medicine, Technion, Haifa 31096, Israel; 6 Oncology Department, Bnai Zion Medical Center, Haifa 3104701, Israel; 7 Edith Wolfson Medical Center, Oncology Institute, Holon 5822012, Israel

**Keywords:** non-small cell lung cancer, brain metastases, epidermal growth factor receptor, anaplastic lymphoma kinase rearrangements, intracranial objective response rate, intracranial disease control rate, overall survival

## Abstract

Background: Brain metastases (BMs) are a common and challenging complication of non-small cell lung cancer (NSCLC), historically associated with a poor prognosis. The development of targeted therapies, specifically tyrosine kinase inhibitors (TKIs) for epidermal growth factor receptor (EGFR) and anaplastic lymphoma kinase (ALK) gene alterations, has significantly improved treatment outcomes. Methods: This article reports and evaluates the efficacy of different generations of TKIs for NSCLC with BMs. The primary endpoints assessed are intracranial objective response rates (IC-ORR), progression-free survival (PFS), and overall survival (OS). The analysis considers TKIs as monotherapy and in combination with radiotherapy. It also examines the impact of newer generation TKIs with enhanced blood–brain barrier (BBB) penetration on intracranial control. The report further discusses the integration of systemic therapy with local modalities like stereotactic radiosurgery (SRS) and the safety profiles of these agents, including central nervous system (CNS) and metabolic adverse events. Results: Newer generation TKIs demonstrate significantly enhanced BBB penetration, resulting in superior intracranial control compared to older generations. These agents show remarkable intracranial activity, contributing to improved IC-ORR, PFS, and OS. The optimal integration of systemic therapy with local modalities, such as SRS, is still under investigation. Treatment with these TKIs is associated with distinct safety profiles, including novel CNS and metabolic adverse events, which require careful management due to prolonged treatment durations. Conclusions: The management of CNS metastases in NSCLC is evolving towards more proactive and personalized therapeutic strategies. Newer generation TKIs have profoundly reshaped the treatment landscape by offering superior intracranial control. Further research is needed to determine the optimal integration of these systemic therapies with local modalities and to effectively manage the associated adverse events.

## 1. Introduction

Lung cancer remains the leading cause of cancer-related death worldwide, with an estimated 2.5 million new cases and 1.8 million deaths in 2022 [1,2]. Lung cancer research has rapidly advanced, moving beyond traditional chemotherapy to a more personalized, targeted approach. Current studies are primarily focused on three major areas: early detection, targeted therapies, and immunotherapy [3,4,5,6,7].

Brain metastases are a prevalent and often devastating complication in patients diagnosed with non-small cell lung cancer, profoundly impacting both prognosis and quality of life. Epidemiological data indicate that approximately 10% to 20% of NSCLC patients present with brain metastases at the time of their initial diagnosis, with a substantial proportion, ranging from 40% to 50%, developing these intracranial lesions over the course of their disease [8,9,10]. Historically, the presence of NSCLC brain metastases has been associated with a notably poor overall survival. These metastatic lesions commonly manifest in specific regions of the brain, with the cerebrum being the most frequent site (80%), followed by the cerebellum (15%), and the brain stem (5%) [11,12,13,14]. The significant burden of brain metastases underscores the critical need for effective therapeutic strategies that can not only control systemic disease but also achieve robust intracranial efficacy [10,11,12,13].

The landscape of NSCLC treatment has been revolutionized by the identification of specific oncogenic driver mutations, such as those in the Epidermal Growth Factor Receptor (EGFR) gene and Anaplastic Lymphoma Kinase (ALK) gene rearrangements. EGFR gene mutations are particularly common in NSCLC patients, observed in approximately 50% of Asian patients and 10% to 20% of Caucasian patients [15,16,17]. A compelling observation in clinical practice is the significantly elevated propensity for brain metastasis development in patients harboring EGFR-sensitive mutations. Studies report that up to 70% of EGFR-positive patients may develop brain metastases, compared to 38% in those with wild-type EGFR. This translates to a stark reality where one in three EGFR-positive NSCLC patients will develop brain metastases during their clinical course [10,11,12,13,14,15].

Similarly, ALK gene rearrangement, found in about 4.5% of NSCLC patients, predominantly those with adenocarcinoma, is strongly associated with central nervous system metastasis. At the time of NSCLC diagnosis, approximately 20% of patients with ALK gene rearrangement already present with brain metastases, and a substantial 40% to 50% of these individuals will develop CNS metastases as their disease progresses [18,19,20,21]. This high incidence of brain involvement in both EGFR-mutated and ALK-rearranged NSCLC highlights a fundamental biological characteristic: these specific driver mutations are not merely oncogenic drivers but also appear to confer an inherent biological advantage to cancer cells for colonizing the central nervous system. This phenomenon suggests that for these patient subsets, CNS surveillance and the proactive implementation of CNS-penetrant therapies are not merely optional but represent an essential component of comprehensive management from the earliest stages of diagnosis. This understanding has profoundly influenced the strategic development of targeted therapies [14,15,16,17,18].

## 2. Materials and Methods

### 2.1. Search Strategy

A systematic literature search was conducted using the PubMed database to identify relevant studies published up to July 2025. The search strategy employed a combination of the following keywords: “non-small cell lung cancer,” “NSCLC,” “brain metastases,” “blood-brain barrier,” “Epidermal Growth Factor Receptor,” “EGFR,” “Anaplastic Lymphoma Kinase,” “ALK,” and “Tyrosine Kinase Inhibitors,” “TKI”.

### 2.2. Study Selection Criteria

Studies were included in the analysis if they met the following criteria:

Patient Population: Individuals with histologically confirmed non-small cell lung cancer (NSCLC) and the presence of brain metastases.

Intervention: Treatment with tyrosine kinase inhibitors (TKIs), including but not limited to gefitinib, erlotinib, afatinib, dacomitinib, osimertinib, crizotinib, alectinib, brigatinib, and lorlatinib.

Reported Outcomes: Studies that reported on safety, intracranial objective response rate (iORR), intracranial disease control rate (iDCR), or overall survival (OS) outcomes.

Case reports, review articles, and studies with insufficient data for extraction were excluded from this analysis.

### 2.3. Data Extraction and Analysis

Two reviewers independently extracted data from the eligible studies to ensure accuracy and consistency. The extracted information included study characteristics, patient demographics, treatment details, and reported outcomes. Pooled response rates and overall survival were calculated and visualized using bar graphs for comparative analysis. Given the anticipated heterogeneity among the included studies, a descriptive synthesis of the findings was performed.

## 3. Challenges of the Blood–Brain Barrier (BBB) in CNS Drug Delivery

A pervasive challenge in the effective pharmacological management of brain metastases has historically been the formidable blood–brain barrier. The BBB, a highly selective semipermeable membrane, severely restricts the passage of many anti-cancer agents into the central nervous system, rendering brain metastases notoriously difficult to treat [15,22,23,24,25,26]. While it is true that in most brain metastases, the BBB is compromised to some extent, this disruption is often heterogeneous and insufficient to allow for effective therapeutic concentrations of many conventional systemic agents. Furthermore, the presence and activity of efflux transporters, such as P-glycoprotein (P-gp), within the BBB actively pump drugs out of the brain, further contributing to inadequate drug concentrations in the CNS [25,26,27,28].

The recognition of the BBB as a primary impediment to effective CNS drug delivery has been a pivotal factor in the evolution of cancer therapeutics. This understanding directly spurred the development of newer generations of TKIs specifically engineered with improved physicochemical properties to enhance their ability to traverse the BBB and achieve therapeutically meaningful concentrations within intracranial lesions [23,24,25,26,27]. This adaptive response in drug design, driven by a deep understanding of the biological barrier, represents a fundamental shift in the approach to managing CNS metastases. It signifies a move from merely treating brain metastases as a late-stage, often isolated, complication to integrating robust CNS control as a primary and proactive goal of systemic targeted therapy from the very outset of treatment [22,23,24,25].

## 4. Efficacy of EGFR-Targeted TKIs in Brain Metastases

The initial generations of EGFR-tyrosine kinase inhibitors, including first-generation agents such as gefitinib, erlotinib, and icotinib, and second-generation TKIs like dacomitinib and afatinib, demonstrated significant systemic activity in EGFR-mutated NSCLC [29,30,31,32]. However, their efficacy against intracranial lesions was notably limited, primarily due to their poor penetration through the blood–brain barrier. The reported cerebral spinal fluid (CSF) to plasma or CSF to blood penetration rates for first-generation TKIs ranged from a mere 1.1% to 3.3%, with second-generation agents showing similarly low rates of approximately 1.7%. Among the first-generation inhibitors, erlotinib exhibited a marginally better BBB penetration capability compared to gefitinib [29,30,31,32].

While some patients experienced transient intracranial tumor shrinkage or alleviation of neurological symptoms upon treatment with these earlier TKIs, the therapeutic effect was generally short-lived and often insufficient for durable control [29,30,31,32,33]. A pooled analysis of studies involving first-generation EGFR-TKIs in NSCLC patients with brain metastases reported an intracranial objective response rate (IC-ORR) of 51.8%, a disease control rate (DCR) of 75.7%, a median progression-free survival (PFS) of 7.4 months, and an overall survival (OS) of 11.9 months. A significant clinical challenge with first-generation TKIs was the development of acquired resistance, frequently mediated by the T790M secondary mutation of the EGFR gene. Interestingly, brain metastases typically do not harbor this T790M mutation, suggesting that the emergence of resistant cancer cells in the CNS was often a consequence of insufficient drug concentration at the site of disease, a phenomenon termed pharmacokinetic resistance [32,33,34,35]. This observation that limited intracranial efficacy persisted despite systemic activity and without the common resistance mutation underscored pharmacokinetic resistance as a major driver of treatment failure in the brain. This critical understanding directly catalyzed the subsequent development of TKI generations specifically engineered for enhanced BBB penetration, demonstrating a clear adaptive response in drug development to overcome this particular mechanism of resistance [33,34,35,36,37,38].

The limitations of earlier generations of EGFR-TKIs, particularly their poor CNS penetration, spurred the development of third-generation agents. Osimertinib, a prominent third-generation EGFR-TKI, was specifically designed to overcome these challenges by exhibiting improved blood–brain barrier penetration, with CSF/plasma or CSF/blood penetration rates ranging from 2.5% to 16.0%. This enhanced CNS activity, coupled with its efficacy against the T790M resistance mutation, has led to a remarkable improvement in intracranial disease control [37,38,39,40,41].

Reported intracranial objective response rates (IC-ORR) for osimertinib have been impressive, ranging from 54% to 71% in some studies, and as high as 76% in others. One study focusing on patients with newly diagnosed, previously untreated brain metastases reported a best objective response rate of 100% at the patient level [38,39,40,41]. At the lesion level, 77.2% of tracked lesions achieved a complete response (CR) as their best objective response. The median overall survival for patients with de novo brain metastases treated with osimertinib was not reached at the time of reporting, with estimated survival rates of 90.9% at 1 year, 79.7% at 2 years, and 62.4% at 3 years [38,39,40,41,42]. Despite these robust responses, intracranial progression can still occur, with reported CNS progression rates of 21% at 1 year, 32% at 2 years, and 41% at 3 years following osimertinib initiation [38,39,40,41]. These data highlight the significant advancements in CNS control achieved with third-generation EGFR-TKIs, making them a cornerstone of treatment for EGFR-mutated NSCLC with brain metastases (Table 1).

### Combination Strategies: EGFR-TKIs with Radiotherapy

**Figure 1:**
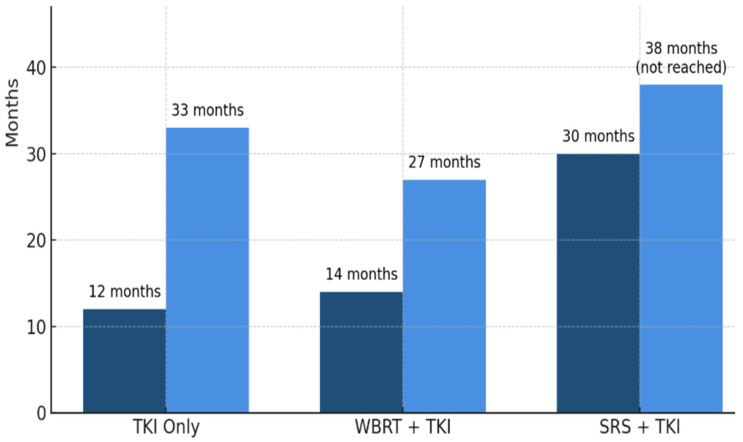
Comparative Overall Survival (OS) and Intracranial Progression-Free Survival (iPFS) for TKI Monotherapy vs. TKI + Radiotherapy in EGFR-Mutated NSCLC Brain Metastases. This figure illustrates the median OS and iPFS for different treatment modalities in EGFR-mutated NSCLC patients with brain metastases. It presents three distinct bars or curves for each endpoint: (1) EGFR-TKI alone (e.g., osimertinib monotherapy), (2) EGFR-TKI combined with Whole Brain Radiation Therapy (WBRT), and (3) EGFR-TKI combined with Stereotactic Radiosurgery (SRS). For instance, the iPFS values show approximately 12 months for TKI-only, 14 months for WBRT + TKI, and 30 months for SRS + TKI. Similarly, OS values reflect median OS not reached for SRS + TKI, 27 months for WBRT + TKI, and 33 months for TKI-only. The graph emphasizes the superior intracranial progression-free survival and overall survival benefits associated with the combination of TKI and SRS, while also showing a comparison of TKI monotherapy versus TKI plus WBRT. Hazard ratios are 0.76 for iPFS and =0.56 for OS for osimertinib + RT vs. osimertinib alone.

The integration of upfront cranial radiotherapy (RT) with EGFR-TKIs in the management of EGFR-mutated NSCLC with brain metastases remains a complex and evolving area of clinical discussion, with an ongoing debate regarding optimal sequencing, especially with newer TKI generations. For patients treated with first-generation EGFR-TKIs, meta-analyses have indicated that upfront brain RT, particularly stereotactic radiosurgery (SRS), significantly improved overall survival (OS). For instance, upfront RT alone showed an OS hazard ratio (HR) of 0.78 (95% CI: 0.65–0.93, *p* = 0.005) compared to TKI alone, and the combination of RT plus TKI demonstrated superior OS (HR = 0.71, 95% CI: 0.58–0.86, *p* = 0.0005) and intracranial PFS (HR = 0.69, 95% CI: 0.49–0.99, *p* = 0.04). Whole brain radiation therapy (WBRT) combined with TKI also appeared to yield better survival outcomes compared to TKI monotherapy. Another meta-analysis further supported that radiotherapy plus EGFR-TKIs was more effective in improving response rate (RR = 1.48) and disease control rate (RR = 1.29), and significantly prolonged the time to CNS progression (HR = 0.56) and median OS (HR = 0.58) when compared to RT alone or RT plus chemotherapy [47,48,49,50,51,52,53]. While osimertinib, a third-generation TKI, has established superior CNS activity as a single agent, a meta-analysis published suggests that combination therapy with upfront brain radiotherapy still offers an improvement in intracranial PFS (HR = 0.76, 95% CI: 0.61–0.94) and overall survival (HR = 0.56, 95% CI: 0.36–0.87) when compared to osimertinib alone [51]. This finding is particularly important as it indicates that even with highly effective systemic agents, the local control provided by radiotherapy, especially SRS, remains a crucial component for optimizing long-term intracranial disease control and patient survival, particularly when considering the potential for delayed neurologic deficits [51]. A retrospective study comparing WBRT + TKI, SRS + TKI, and TKI-only in EGFR-mutated NSCLC with brain metastases provided further clarity. This study found that the SRS + TKI approach significantly improved intracranial PFS (median 30 months) compared to WBRT + TKI (median 14 months) and TKI-only (median 12 months). Furthermore, overall survival was significantly longer in the SRS + TKI group (median not reached) compared to WBRT + TKI (median 27 months) and TKI-only (median 33 months). This suggests that SRS combined with TKI may represent an optimal strategy for intracranial control, regardless of the TKI generation employed. The consistent observation of benefit with local therapy, especially SRS, even in the era of highly CNS-penetrant TKIs, challenges the initial assumption that superior systemic CNS penetration alone would negate the need for local treatment, highlighting the intricate balance required in managing brain metastases (Figure 1) [52,53,54,55,56].

## 5. Efficacy of ALK-Targeted TKIs in Brain Metastases

Crizotinib, the first ALK inhibitor approved for ALK-positive NSCLC, marked a significant advance in systemic disease control. However, its therapeutic impact on brain metastases was considerably limited due to its poor penetration across the blood–brain barrier. Consequently, progression within the central nervous system frequently emerged as the primary manifestation of treatment failure for patients receiving crizotinib [57,58,59,60]. A retrospective study highlighted this vulnerability, revealing that a substantial 72% of patients with pre-existing brain metastases who were treated with crizotinib subsequently experienced secondary CNS progression [60].

Clinical trial data further illustrate these limitations. In the ALEX trial, the intracranial treatment response rate for crizotinib was reported at 40% in patients who had not received prior local treatment to the brain, and 71.4% in those who had undergone prior irradiation. The median PFS specifically for patients presenting with primary CNS metastases and treated with crizotinib was a modest 7.4 months. These outcomes underscored the urgent need for ALK inhibitors with enhanced CNS penetration to effectively address the high propensity for brain metastasis in ALK-positive NSCLC [61,62,63].

The clinical challenges posed by crizotinib’s limited CNS penetration led to the development of second-generation ALK-TKIs, including alectinib, ceritinib, and brigatinib. These agents were specifically engineered with improved blood–brain barrier penetration capabilities to overcome crizotinib resistance and enhance intracranial activity [64,65].

Alectinib has demonstrated clear superiority over crizotinib in terms of intracranial efficacy. In the pivotal ALEX trial, the rate of CNS progression at 12 months was significantly lower in the alectinib group (12%) compared to the crizotinib group (45%), with a hazard ratio of 0.16 (*p* < 0.001). Intracranial objective response rates (IC-ORR) for alectinib were reported at 78.6% in patients without prior local radiotherapy and an impressive 85.7% in those who had received prior irradiation [61]. Furthermore, alectinib significantly prolonged intracranial median progression-free survival (mPFS) to 36.0 months, a substantial improvement compared to 10.8 months with crizotinib (*p* < 0.001) [61]. The median overall PFS for alectinib was 27.5 months, versus 9.5 months for crizotinib. Despite these superior intracranial and systemic PFS benefits, studies have not consistently shown a significant difference in overall survival between alectinib and crizotinib (median OS not reached for alectinib vs. 58.7 months for crizotinib, *p* = 0.149). This observation may be influenced by factors such as patient crossover to second-generation TKIs after crizotinib progression or the duration of follow-up [61].

While ceritinib and brigatinib also demonstrated improved CNS penetration compared to crizotinib, detailed comparative intracranial efficacy data from meta-analyses within the provided literature are less comprehensive than for alectinib and lorlatinib. Nevertheless, these second-generation agents are generally recognized for their enhanced ability to control CNS disease relative to crizotinib [64,65,66,67].

The development of third-generation ALK-TKIs represents a significant advancement in the management of ALK-positive NSCLC, particularly for brain metastases. Lorlatinib, a prime example of this class, possesses a unique macrocyclic chemical structure that ensures excellent brain penetration and maintains activity against a broad spectrum of resistance mutations that can emerge with earlier-generation ALK inhibitors [65,66,67,68].

The pivotal Phase III CROWN trial, which compared lorlatinib to crizotinib as first-line treatment for advanced ALK-positive NSCLC, yielded remarkable results in both systemic and intracranial efficacy [68]. The median progression-free survival (PFS) for lorlatinib was not reached, a stark contrast to 9.1 months for crizotinib, translating to a hazard ratio of 0.19 (95% CI: 0.13–0.27). The 5-year PFS rate was an impressive 60% for lorlatinib, compared to only 8% for crizotinib, indicating highly durable systemic control.

The intracranial efficacy of lorlatinib was particularly striking. The median time to intracranial progression was not reached for lorlatinib, while it was 16.4 months for crizotinib (HR = 0.06, 95% CI: 0.03–0.12). The intracranial objective response rate (IC-ORR) for lorlatinib was 60%, with 49% of patients achieving complete responses in brain lesions. Furthermore, the CROWN trial demonstrated a remarkable ability of lorlatinib to prevent new brain lesions: 92% (95% CI: 85–96%) of patients did not develop intracranial progression over five years, and 83% of those with brain metastases at baseline remained progression-free at 5 years. Among patients without baseline brain metastases (n = 114), only four patients developed brain metastases by investigator assessment. This data indicates that for patients with brain metastases at baseline, lorlatinib conferred the best PFS among ALK inhibitors [68].

The observation of a “plateau” in the progression-free survival curve for lorlatinib, with a 5-year PFS rate of 60%, is highly significant. In oncology, a plateau in survival curves often suggests that a subset of patients may achieve durable long-term control or even functional cure, rather than merely delayed progression. This is an emerging pattern that raises questions about whether some patients are becoming “long-responders”, akin to those observed with certain immunotherapies. This phenomenon has profound implications for patient counseling and the development of long-term management strategies, suggesting a potential shift in the disease trajectory for a subset of patients. The ability of lorlatinib to not only treat existing brain metastases but also actively prevent the formation of new ones represents a new paradigm in CNS control. This represents a significant shift from a reactive treatment approach, which addresses established metastases, to a proactive strategy focused on preventing CNS progression, fundamentally altering the natural history of the disease in the brain (Table 2) [68,69,70,71].

### Combination Strategies: ALK-TKIs with Radiotherapy

The role of combining ALK-TKIs with radiotherapy for brain metastases in ALK-positive NSCLC is also an area of active investigation, mirroring the discussions in EGFR-mutated disease. Some retrospective studies have suggested that the integration of targeted therapy with intracranial radiation may improve both intracranial progression-free survival and overall survival in ALK-positive NSCLC patients with brain metastases [74,75,76,77].

However, the evidence regarding combination therapy is not entirely consistent. One meta-analysis, encompassing both ALK and EGFR mutations, reported no significant difference in median overall survival or progression-free survival when comparing TKIs combined with radiotherapy, radiotherapy alone, or TKIs alone. Specifically, this analysis found no significant progression-free survival difference between whole brain radiation therapy (WBRT) plus TKI versus WBRT alone, or stereotactic radiosurgery (SRS) plus TKI versus SRS alone [74,75,76,77,78]. This apparent contradiction with other findings underscores the heterogeneity in study designs, patient populations, and TKI generations included in different analyses. It highlights the complexity of determining the optimal sequencing and combination strategies, suggesting that treatment decisions often require careful individualization based on lesion characteristics, patient symptoms, and the specific TKI being utilized [74,75,76,77,78,79].

## 6. Comparative Efficacy and Clinical Implications

### 6.1. Cross-Generational and Cross-Mutation Efficacy Comparisons

The available evidence reveals a consistent and compelling trend, across both EGFR and ALK mutations, that the intracranial efficacy of tyrosine kinase inhibitors has progressively improved with each successive generation. This enhancement is primarily attributable to advancements in drug design that facilitate superior penetration of the blood–brain barrier [29,30,31,32,57,58,59].

First and second-generation TKIs, while effective systemically, demonstrated limited CNS penetration (e.g., 1.1–3.3% CSF/plasma ratio for first-generation EGFR-TKIs and poor for crizotinib in ALK-positive disease), leading to suboptimal intracranial control [57,58,59]. In contrast, third-generation TKIs, exemplified by osimertinib for EGFR-mutated NSCLC and lorlatinib for ALK-rearranged disease, represent the current benchmark for intracranial disease management [69,70,71]. These agents exhibit significantly higher BBB penetration (e.g., 2.5–16.0% for osimertinib) and have achieved unprecedented intracranial objective response rates and substantially prolonged intracranial progression-free survival [45,46]. Lorlatinib, in particular, has demonstrated remarkable long-term CNS control and a notable ability to prevent the formation of new brain lesions, a critical development in the field. The overall survival benefits have also been observed with these newer generations, although the precise role and timing of local therapy in combination with these highly effective systemic agents remain a nuanced and actively debated topic [69,70,71].

### 6.2. Impact of BBB Penetration on Clinical Outcomes

The ability of a TKI to efficiently penetrate the blood–brain barrier and achieve therapeutic concentrations within the central nervous system is a paramount determinant of its intracranial efficacy. The historical challenge of the BBB, which limited the entry of many conventional anti-cancer drugs, directly led to the design and development of newer TKI generations with enhanced CNS permeability. These newer agents were specifically modified to be less susceptible to efflux transporters, such as P-glycoprotein, thereby increasing their concentration within brain lesions [25,26,27,28].

The clinical data consistently demonstrate a direct correlation between improved BBB penetration and superior intracranial response rates, as well as prolonged intracranial progression-free survival. This relationship is a consistent theme observed across both EGFR and ALK pathways [29,30,31,32,63,64,65]. For instance, the limited CNS activity of first-generation EGFR-TKIs and crizotinib was directly linked to their poor BBB penetration, often resulting in CNS progression as the primary site of treatment failure despite systemic control. Conversely, the remarkable intracranial efficacy of osimertinib and lorlatinib is a direct consequence of their optimized pharmacokinetic properties, allowing them to effectively target and control intracranial disease. This fundamental understanding of drug pharmacokinetics in the CNS has been instrumental in shaping the current therapeutic landscape for NSCLC brain metastases [6,7,8,9,10].

### 6.3. Guiding Treatment Decisions for NSCLC Brain Metastases

The presence and characteristics of brain metastases are critical considerations when selecting the most appropriate TKI therapy, given the varying intracranial activity of different agents and generations. For asymptomatic patients with EGFR-mutated brain metastases, the high CNS activity of third-generation agents like osimertinib suggests that upfront CNS-active systemic therapy alone may be a reasonable initial approach [15,16,17]. However, the optimal first-line strategy, specifically whether to rely solely on osimertinib or to integrate upfront local therapy, remains a subject of considerable discussion and warrants further prospective clinical trials to provide definitive guidance [32,33,39].

For ALK-positive patients, the superior intracranial efficacy demonstrated by second- and third-generation ALK-TKIs, such as alectinib and lorlatinib, strongly supports their preferential use for individuals who present with brain metastases or are at high risk of developing them [64,65,66].

The data concerning combination therapy with radiotherapy present a more complex picture, with findings that are sometimes contradictory. While some studies indicate a benefit for upfront radiotherapy, particularly stereotactic radiosurgery (SRS), in combination with TKIs to optimize intracranial control and overall survival, other meta-analyses have not found a significant difference in survival outcomes when TKIs are added to radiotherapy or used alone [49,50,51,52]. This discrepancy in outcomes is a critical point that warrants careful consideration. It suggests that differences in study design, including patient selection criteria, the specific TKI generation employed, the modality of radiotherapy (WBRT vs. SRS), the chosen endpoints, and the duration of follow-up, significantly influence the reported results [74,75,76,77,78,79]. This highlights a need for more harmonized methodologies in future research, perhaps through direct head-to-head prospective trials comparing TKI monotherapy versus TKI plus radiotherapy (especially SRS) for specific TKI generations. Such trials are essential to provide definitive clinical guidance and resolve these inconsistencies [47,48,49,75,77].

The historical cornerstone of brain metastasis management was radiotherapy. With the advent of first and second-generation TKIs, their limited BBB penetration meant that radiotherapy remained a critical component of treatment [74,75]. However, the emergence of highly CNS-penetrant third-generation TKIs has created a paradigm shift, where systemic therapy alone can achieve significant intracranial control [47,48,49,50]. The ongoing discussion about the necessity of upfront radiotherapy, even with highly effective agents like osimertinib, signifies a complex clinical decision point [55]. This indicates that the “standard of care” is no longer a monolithic approach but a dynamic, personalized strategy that must carefully balance systemic efficacy, local control, and the associated toxicity profiles to optimize patient outcomes. This evolving definition of standard of care necessitates a highly individualized treatment plan based on factors such as the number and size of lesions, the presence and severity of symptoms, and the patient’s overall performance status [55,56].

## 7. Safety Profile and Adverse Events of TKIs in Brain Metastases

### 7.1. Adverse Events Associated with EGFR-TKIs

Tyrosine kinase inhibitors targeting EGFR mutations are generally associated with a characteristic spectrum of adverse events. For first-generation EGFR-TKIs such as gefitinib and erlotinib, common drug-related toxicities include rash, observed in 62.8% of patients, diarrhea (38.5%), fatigue (27.7%), hepatotoxicity (20.9%), nausea (16.9%), and vomiting (4.7%) [5,6,15,16,17]. The majority of these adverse events are typically mild to moderate in intensity (Grade 1–2). More severe toxicities (Grade 3–4) occurred in 11.5% of patients, primarily manifesting as severe rash, hepatotoxicity, and diarrhea. These severe events were often manageable with dose reductions, and notably, no patient in one study had to discontinue EGFR-TKI treatment due to side effects. Furthermore, interstitial lung disease, a serious but rare complication, was not reported in the particular study [5,6,80,81]. The overall safety profile of these EGFR-TKIs in patients with brain metastases is consistent with their known tolerability in broader NSCLC populations, indicating that the presence of brain lesions does not significantly alter the systemic toxicity profile [5,6,7,8,9].

### 7.2. Adverse Events Associated with ALK-TKIs

While ALK inhibitors have significantly improved the prognosis for patients with ALK-positive NSCLC, their use is associated with a distinct set of adverse events, including serious adverse events (SAEs) that warrant careful attention [66,67,68,69,70,71,72,73,74,75,76]. The overall incidence of SAEs varies among different ALK inhibitors, with alectinib generally appearing to be the safest (overall SAE incidence of 26.24%), whereas ceritinib (41.44%) and brigatinib (41.68%) are associated with higher rates of SAEs.

For crizotinib, the overall SAE incidence was 38.09%. Common SAEs included pneumonia (4.21%), thrombotic disease (3.71%), and pleural effusion (1.26%). Alectinib, while generally safer, had pneumonia as its highest incidence SAE (0.80%), with other SAEs occurring at very low rates. Ceritinib showed a higher incidence of nervous system toxicity (8.84%), dyspnea/respiratory failure (5.50%), pneumonia (4.29%), and notable alimentary SAEs such as nausea, vomiting, and diarrhea. Brigatinib was associated with significant lung toxicity, including pneumonia (5.09%) and dyspnea/respiratory failure (4.29%), along with a higher incidence of nervous system toxicity (7.40%) [66,67,68,69].

Lorlatinib, a third-generation ALK-TKI, possesses a unique safety profile that can present management challenges. A high incidence of metabolic syndrome is characteristic, with hypercholesterolemia reported in 72% of patients (21.5% Grade ≥ 3) and hypertriglyceridemia in 66% (17% Grade 3, 8% Grade 4). Hypolipidemic treatment is frequently recommended for managing these metabolic changes. Furthermore, neurocognitive adverse events (NAEs) are a notable feature of lorlatinib, including cognitive effects (14.57%), mood effects (11.17%), speech changes (7.24%), and psychotic effects (4.97%). Approximately 40% of patients in the CROWN study experienced NAEs, though most were Grade 1 or 2. Overall, Grade ≥ 3 treatment-related adverse events were reported in 66% of patients in the CROWN trial [68,69,70,71].

**Figure 2:**
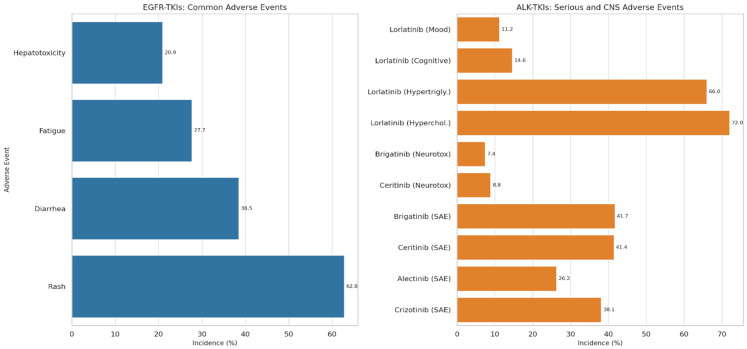
Incidence of Adverse Events Associated with Major EGFR and ALK Tyrosine Kinase Inhibitors. This conceptual graph compares the incidence of common and serious adverse events across representative EGFR and ALK TKIs. For EGFR-TKIs, it shows percentages for rash (e.g., 62.8%), diarrhea (e.g., 38.5%), fatigue (e.g., 27.7%), and hepatotoxicity (e.g., 20.9%). For ALK-TKIs, it presents the overall SAE incidence for crizotinib (e.g., 38.09%), alectinib (e.g., 26.24%), ceritinib (e.g., 41.44%), and brigatinib (e.g., 41.68%). Additionally, it highlights specific CNS-related AEs for ceritinib (nervous system toxicity 8.84%) and brigatinib (nervous system toxicity 7.40%), and for lorlatinib, it shows the incidence of hypercholesterolemia (e.g., 72%), hypertriglyceridemia (e.g., 66%), and neurocognitive effects (e.g., cognitive 14.57%, mood 11.17%).

As TKIs, particularly the newer generations, demonstrate enhanced ability to penetrate the blood–brain barrier, their toxicity profiles also evolve, with the emergence of unique CNS-related adverse events [66]. For instance, the higher nervous system toxicity observed with second-generation ALK-TKIs like ceritinib and brigatinib, and the distinct spectrum of neurocognitive and metabolic adverse events with lorlatinib, illustrate this pattern [66,67,74]. This implies a clinical trade-off: improved CNS efficacy is accompanied by a new set of CNS-specific toxicities that clinicians must be prepared to identify and manage, moving beyond the more common dermatologic and gastrointestinal adverse events associated with earlier TKIs [72,73,74]. Given that patients on highly effective TKIs, especially third-generation agents like lorlatinib, may remain on treatment for years, the long-term management of these specific adverse events becomes paramount. The high incidence of metabolic syndrome with lorlatinib, for example, necessitates proactive monitoring and the initiation of hypolipidemic treatment [68,69,70,71]. This highlights that successful TKI therapy extends beyond mere tumor control to encompass comprehensive patient management, including diligent monitoring for and early intervention of chronic, drug-specific toxicities to ensure sustained patient quality of life and adherence to treatment (Figure 2).

### 7.3. Safety Considerations for TKI-Radiotherapy Combinations

The combination of tyrosine kinase inhibitors with radiotherapy for brain metastases introduces specific safety considerations. Meta-analyses indicate that while this combined approach can yield superior efficacy, it is also associated with a higher overall incidence of adverse events compared to radiotherapy or TKI monotherapy alone. For instance, the combination of radiotherapy and EGFR-TKIs has been shown to result in an increased overall incidence of adverse events (Risk Ratio (RR) = 1.25, 95% CI: 1.01–1.57, *p* = 0.009) [52].

Specific adverse events that are significantly more common in the combined treatment group include rash (RR = 4.97, 95% CI: 2.68–9.21, *p* = 0.000) and dry skin (RR = 8.44, 95% CI: 1.48–48.28, *p* = 0.017) [52]. However, other common TKI-related adverse events, such as fatigue, nausea/vomiting, and diarrhea, were largely mild and tolerable, with no significant difference in incidence compared to control groups. Pneumonitis, a serious lung toxicity, was rarely observed in these combination settings [52]. Notably, recent data suggest that the combination of osimertinib with upfront brain radiotherapy demonstrates an acceptable safety profile, indicating that the benefits of enhanced intracranial control can be achieved without an undue increase in toxicity burden [52]. These findings emphasize the importance of anticipating and managing specific dermatologic toxicities when combining TKIs with cranial radiotherapy, while generally reassuring clinicians about the overall tolerability of this integrated approach.

## 8. Drug Resistance Mechanisms and Challenges in CNS Metastases of Lung Cancer

CNS metastases remain a major therapeutic challenge in lung cancer, particularly in NSCLC. Despite advances in targeted therapy and immunotherapy, intracranial control is often limited due to unique resistance mechanisms and biological barriers.

Drug Resistance Mechanisms

1. Blood–Brain Barrier (BBB) and Blood–Tumor Barrier (BTB):
  The BBB restricts the entry of most chemotherapeutic agents and targeted therapies into the CNS through tight endothelial junctions, efflux pumps such as P-glycoprotein, and enzymatic degradation. Even in established brain metastases, the BTB remains heterogeneous, creating areas of poor drug penetration [82,83].
2. Tumor Cell–Intrinsic Mechanisms:
  Secondary mutations (e.g., EGFR T790M, C797S; ALK G1202R) reduce the binding affinity of TKIs [84].
  Bypass signaling activation (e.g., MET amplification, HER2 alterations, KRAS mutations) can restore downstream signaling despite receptor inhibition [85].
  Intratumoral and interlesional heterogeneity between CNS and extracranial disease leads to variable treatment responses [86].
3. Microenvironmental Resistance:
  The CNS microenvironment, including astrocytes and microglia, can secrete cytokines such as IL-6 and TGF-β, promoting tumor survival, stemness, and drug tolerance. Hypoxia and metabolic reprogramming further impair drug sensitivity [87].
4. Immune-Mediated Resistance:
  The CNS is relatively immune-privileged, with reduced T-cell infiltration and impaired antigen presentation. PD-L1 expression and T-cell exhaustion within brain metastases limit the efficacy of ICIs [88].
5. Pharmacokinetic and Pharmacodynamic Barriers:
  Inadequate solubility, rapid clearance, or efflux-mediated exclusion of drugs prevents therapeutic concentrations from being sustained within intracranial lesions, even when systemic disease remains controlled [89].

## 9. Discussion

The therapeutic landscape for brain metastases in EGFR and ALK-mutated non-small cell lung cancer has been fundamentally transformed by the advent and evolution of tyrosine kinase inhibitors. Successive generations of TKIs have demonstrated significantly improved central nervous system penetration, directly translating into enhanced intracranial efficacy [7,8]. Third-generation TKIs, exemplified by osimertinib for EGFR-mutated disease and lorlatinib for ALK-rearranged NSCLC, currently represent the pinnacle of intracranial control, achieving superior objective response rates and remarkably prolonged intracranial progression-free survival [40,68]. Lorlatinib, in particular, has shown unprecedented long-term CNS control and a notable capacity to prevent the formation of new brain lesions, marking a significant shift in the management paradigm [71].

The role of combination therapy involving TKIs and radiotherapy remains a nuanced and evolving discussion. While evidence suggests benefits for upfront local radiotherapy, especially stereotactic radiosurgery, even with highly CNS-penetrant TKIs, to optimize intracranial control and overall survival, the precise integration varies by TKI generation and clinical context [49,50,51,80]. Each TKI generation and specific drug is associated with a distinct safety profile. Newer agents, while offering superior efficacy, introduce novel CNS and metabolic adverse events that necessitate specialized management strategies to ensure patient well-being during prolonged treatment durations [66,67,68,69,70,71].

Despite the remarkable progress, several critical unmet needs and avenues for future research persist in the management of NSCLC brain metastases.

One primary area requiring further investigation is the optimal sequencing and combination strategies of TKIs and radiotherapy. While current data offer guidance, definitive prospective trials are needed to establish the most effective integration of TKIs with various radiotherapy modalities (e.g., SRS versus WBRT) across diverse clinical scenarios [52,53,54,55,56]. These scenarios include patients with asymptomatic versus symptomatic brain metastases, varying numbers and sizes of lesions, and specific TKI generations. The conflicting findings from existing meta-analyses regarding combination therapy underscore the necessity for more harmonized methodologies and direct comparative studies to provide clear, actionable guidance [56].

Another crucial area is the management of acquired resistance. While newer TKIs effectively address common resistance mechanisms to earlier generations, understanding and ultimately overcoming resistance to frontline third-generation TKIs remains an active and essential area of research. The long-term efficacy of these agents is impressive, but disease progression will eventually occur, necessitating strategies for subsequent lines of therapy [90,91].

Given the enhanced CNS penetration and the emergence of specific neurocognitive adverse events associated with newer TKIs, long-term studies on cognitive function and overall quality of life are critical. As patients live longer with brain metastases due to effective TKI therapy, the balance between treatment efficacy, the specific adverse event profiles of these drugs (especially novel CNS and metabolic toxicities), and overall quality of life becomes increasingly important for long-term management [52,53,54,55,56]. This necessitates a holistic approach to patient care, emphasizing not just tumor shrinkage but also proactive management of side effects to ensure sustained patient well-being and adherence to treatment over years.

Furthermore, research into personalized treatment approaches is gaining momentum. The identification and validation of biomarkers, such as those derived from MRI radiomics, that can predict the intracranial efficacy of specific TKIs could significantly help in tailoring treatment strategies to individual patient characteristics and tumor biology [22,34]. This level of precision medicine holds promise for optimizing outcomes and minimizing unnecessary toxicities.

Finally, addressing health disparities remains an ongoing concern. Ensuring equitable access to these advanced and often costly therapies and effectively managing the financial toxicity associated with long-term treatment for patients with CNS tumors, are critical aspects of comprehensive cancer care that require continued attention and systemic solutions [22,34].

The evolution of TKIs has moved the goal of treatment for CNS metastases beyond merely alleviating symptoms or delaying progression to potentially achieving long-term disease control or even eradication in the brain, as evidenced by the “plateau” in PFS curves and high rates of non-progression with agents like lorlatinib [71]. This represents a profound shift in clinical aspiration, transforming a historically terminal diagnosis into a potentially manageable chronic condition for a significant subset of patients. The continued advancements in TKI therapy, coupled with a deeper understanding of their CNS activity and toxicity, will undoubtedly continue to reshape the future of NSCLC brain metastasis management, emphasizing an integrated, multidisciplinary, and patient-centric approach.

## 10. Conclusions

EGFR and ALK TKIs have significantly improved outcomes for NSCLC patients with brain metastases, offering effective intracranial control with reduced reliance on radiation therapy. Third-generation EGFR TKIs like osimertinib demonstrate high CNS penetration and IC-ORR up to 76–100%, with prolonged IC-PFS and OS, especially in treatment-naïve patients. Similarly, next-generation ALK TKIs such as alectinib, brigatinib, and lorlatinib have shown substantial CNS efficacy. Alectinib and brigatinib achieve IC-ORR rates above 75% and IC-PFS of over 24–36 months, while lorlatinib, with its superior blood–brain barrier penetration, achieves complete responses in nearly half of patients with CNS involvement. These advances have made targeted therapies the preferred first-line treatment in ALK- and EGFR-positive NSCLC with brain metastases, establishing a paradigm shift toward systemic CNS-directed therapy.

## Figures and Tables

**Table 1:** Comparison of Intracranial Efficacy Metrics of EGFR-Targeted TKIs by Generation.

TKI Generation/Drug	Blood–Brain Barrier Penetration (CSF/Plasma Ratio)	Intracranial Objective Response Rate	Median Intracranial Progression-Free Survival	Median Overall Survival	Reference
First Generation (Gefitinib/Erlotinib)	1.1–3.3%	~50–60% (51.8%)	~7.4 months	~11.9 months	[42]
Second Generation (Afatinib/Dacomitinib)	~1.7%	Variable/Limited	Not consistently reported	Not consistently reported	[43,44]
Third Generation (Osimertinib)	2.5–16.0%	54–100% (commonly ~76%)	~8–15 months (varies by study)	Not reached (especially in de novo brain metastases)	[45,46]

Abbreviation: Cerebrospinal fluid, CSF.

**Table 2:** Comparison of Intracranial Efficacy Metrics of ALK-Targeted TKIs by Generation.

TKI Generation/Drug	Blood–Brain Barrier Penetration	Intracranial Objective Response Rate	Median Intracranial Progression-Free Survival	Median Overall Survival	References
First Generation (Crizotinib)	Poor	~40% (no prior radiotherapy), ~70% (with prior radiotherapy)	~10.8 months	~58.7 months (selected subgroups)	[72]
Second Generation (Alectinib)	Improved	~78.6% (no prior radiotherapy), ~85.7% (with prior radiotherapy)	~36.0 months	Not reached	[61,73]
Second Generation (Brigatinib)	Good	~78% (treatment-naïve patients)	~24.0 months	Not reached	[67,74]
Third Generation (Lorlatinib)	Excellent	~60%, ~49% complete response	Not reached (in first line)	Not evaluable/immature data	[68,69]

## Data Availability

The original contributions presented in this study are included in the article. Further inquiries can be directed to the corresponding author.

## Abbreviations

The following abbreviations are used in this manuscript:

ALK	Anaplastic lymphoma kinase
BBB	Blood–brain barrier
BMs	Brain metastases
CNS	Central nervous system
DCR	Disease control rate
EGFR	Epidermal growth factor receptor
IC-ORR	Intracranial objective response rates
iDCR	Intracranial disease control rate
iORR	Intracranial objective response rate
iPFS	Intracranial progression-free survival
NSCLC	Non-small cell lung cancer
OS	Overall survival
PFS	Progression-free survival
SRS	Stereotactic radiosurgery
TKIs	Tyrosine kinase inhibitors
